# Chimaeric plant-produced bluetongue virus particles as potential vaccine candidates

**DOI:** 10.1007/s00705-023-05790-x

**Published:** 2023-06-13

**Authors:** A. Gwynn, S. Mbewana, B. A. Lubisi, H. M. Tshabalala, E. P. Rybicki, A. E. Meyers

**Affiliations:** 1 Biopharming Research Unit, Department of Molecular and Cell Biology, University of Cape Town, Rondebosch, 7700 South Africa; 2 Diagnostic Services Programme, ARC-Onderstepoort Veterinary Research Institute, Pretoria, 0110 South Africa; 3 Institute of Infectious Disease and Molecular Medicine, Faculty of Health Sciences, University of Cape Town, Observatory, CapeTown, 7925 South Africa

## Abstract

Bluetongue virus (BTV) causes bluetongue disease in ruminants and sheep. The current live attenuated and inactivated vaccines available for prevention pose several risks, and there is thus a need for vaccines that are safer, economically viable, and effective against multiple circulating serotypes. This work describes the development of recombinant virus-like particle (VLP) vaccine candidates in plants, which are assembled by co-expression of the four BTV serotype 8 major structural proteins. We show that substitution of a neutralising tip domain of BTV8 VP2 with that of BTV1 VP2 resulted in the assembly of VLPs that stimulated serotype-specific antibodies as well as virus-specific neutralising antibodies.

**Supplementary Information:**

The online version contains supplementary material available at 10.1007/s00705-023-05790-x.

Bluetongue virus (BTV) is a non-enveloped dsRNA virus belonging to the family *Sedoreoviridae*, genus *Orbivirus*, that typically infects ruminants and causes severe haemorrhagic disease in sheep [1]. The eradication and control of BTV is a challenge due to the existence of 24 classical and several other atypical serotypes as well as their limited serological cross-reactivity [2]. Serotypes are determined by the variability of segment 2 of the genome, encoding the VP2 structural protein, which elicits a neutralising antibody response. During infection, the tip domain of VP2 points outwards from the virion, and binding of VP2-specific antibodies can induce serotype-specific neutralisation [3]. South Africa has an extensive diversity of BTV, with at least 21 serotypes in circulation [4]. Currently, vaccination using a modified live virus (MLV) vaccine or an inactivated vaccine is the most effective method of controlling BTV spread and preventing severe disease. The MLV regime consists of three doses containing five serotypes each, which can be onerous to administer. The inactivated vaccine is a safer alternative than the MLV, as there are no risks of reassortment of vaccine and wild isolates or reversion to virulence. However, the short-lived immunogenicity of inactivated vaccines has encouraged the development of alternative candidates that are safer and more cost-effective, are efficacious for a longer period, and are protective against multiple serotypes.

A potentially very suitable vaccine regime would be the use of virus-like particles (VLPs). These mimic the protein shell of the native virion but lack any genetic material, making them non-infectious and unable to undergo replication or cause viraemia [5]. BTV VLPs are assembled by co-expression of the four major structural viral proteins – VP2, VP3, VP5 and VP7 – and several research groups have produced BTV VLPs representing different serotypes using a baculovirus/insect cell expression system [6]. Despite their immunogenicity and efficacy, however, this production system is insufficiently cost-effective to compete with the commercially available inactivated vaccines [6]. The use of plants as a production platform, however, has the appeal of low complexity of the upstream process, far greater ease of scale-up, and lower operating costs with no need for upstream sterility. Moreover, there are numerous published examples of plant-made vaccines developed for veterinary use [7]. We have previously shown that plant-produced BTV8 VLPs elicited serotype-specific antibodies in sheep, which protected them against BTV8 challenge [8]. Furthermore, Mokoena et al. showed that plant-produced double chimaeric BTV VLPs targeting serotypes 3 and 4 induced seroconversion in sheep [9].

The requirement for co-expression of all four full-length serotype-specific BTV structural proteins to make BTV VLPs targeting all or most of the serotypes is cumbersome. In addition, Thuenemann [10] reported that BTV10 VLPs do not easily form stable VLPs to the same extent that the BTV8 VLPs do. Similarly, we have shown in the laboratory that VLPs representing other BTV serotypes such as 2 and 6 are not as easily assembled using the plant expression platform due to much lower or even undetectable amounts of the component proteins (data not shown). Accordingly, since BTV8 VLPs are readily assembled in plants, we investigated a streamlined approach to use them as a backbone, substituting only the immunogenic “tip” domain of BTV8 VP2 with that of the corresponding domain of a different serotype – in this case BTV1 to generate a chimaeric VP2. Co-expression of BTV8 VP3, 5, and 7 and the chimaeric VP2 in plants resulted in chimaeric BTV1/8 VLPs, which were used to immunise guinea pigs to test their immunogenicity and evaluate their capability of eliciting neutralising antibodies.

The BTV1/8 chimaeric *VP2* gene was synthesised by GenScript (USA). A consensus sequence for the BTV1 VP2 tip domain (base pairs 579-1241), created by aligning BTV1 VP2 genes from 37 different BTV1 strains available at the time in GenBank (Supplementary Table S1), was human-codon-optimised (to maximise expressed protein levels [11]) and substituted in the corresponding region of BTV8 VP2 (*BTV1/8VP2*) (Fig. 1A). AgeI and XhoI restriction enzyme sites at the N- and C- termini, respectively, of *BTV1/8VP2* facilitated cloning into the pEAQ-*HT* vector to generate chimaeric pEAQ-*HT*-BTV1/8 VP2. One hundred ng of pEAQ-*HT*-BTV1/8 VP2 was introduced by electroporation into *Rhizobium radiobacter* AGL-1 cells (ATCC BAA-101). Transformed colonies from cells plated on LB agar supplemented with carbenicillin (25 µg/mL) and kanamycin (30 µg/mL) and incubated at 27°C for 2 days were screened by PCR to confirm the presence of pEAQ-*HT*-BTV1/8 VP2.

**Fig. 1:**
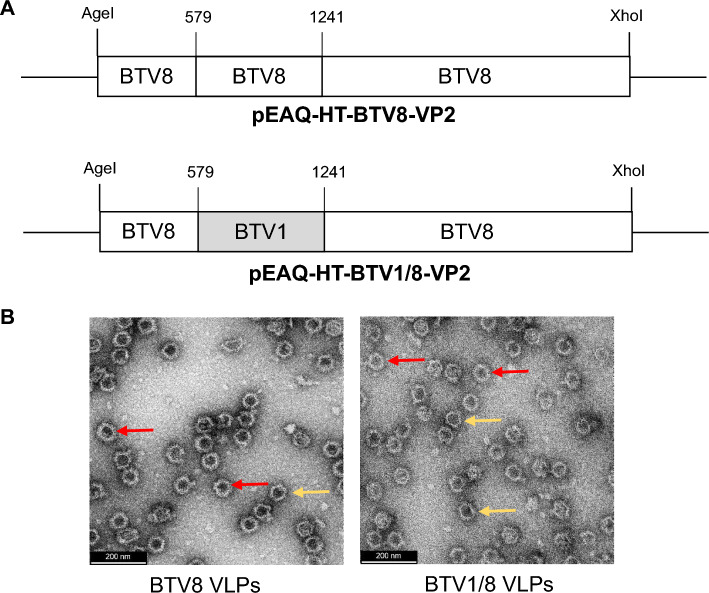
BTV virus-like particle (VLP) assembly using wild-type and chimaeric VP2 constructs. (A) Design of VP2 encoded by pEAQ-HT-BTV8 VP2 and pEAQ-HT-BTV1/8 VP2. BTV1/8 VP2 was synthesised by substitution of nucleotides 579 to 1241 of BTV8 VP2 encoding the VP2 tip domain with those of a consensus sequence of BTV1 VP2. (B) TEM of fraction 8 of plant-produced BTV8 and BTV1/8 VLPs purified on Optiprep™ density gradients. Yellow arrows indicate CLPs. Red arrows indicate VLPs. Scale bars represent 200 nm.

For infiltration, cells harbouring constructs encoding BTV8 proteins (pEAQ-HT-BTV8 VP2, VP3, VP5, and VP7) described previously [8] (Fig. 1A) and *R. radiobacter* harbouring pEAQ-*HT*-BTV1/8 VP2 were cultured in LB broth supplemented with appropriate antibiotics overnight at 27°C overnight with agitation. For co-infiltration, cell cultures containing each construct were adjusted to an OD_600_ of 0.5 in infiltration medium as described previously [11]. Combined cultures harbouring constructs encoding either all four BTV8 VPs or chimaeric BTV1/8 VP2 and BTV8 VP3, VP5, and VP7 were vacuum-infiltrated into whole *Nicotiana benthamiana* plants and incubated as described previously [12]. Plants similarly infiltrated with a culture harbouring pEAQ-*HT* lacking any gene of interest served as negative controls.

Approximately 25-30 g of leaf material was harvested from infiltrated plants at 5 days post-infiltration (dpi) and homogenised using an IKA® T25 digital ULTRA-TURRAX tissue disperser in 2 volumes of bicine buffer (50 mM bicine, 400 mM NaCl, pH 9) containing 1X Roche® EDTA-free complete protease inhibitor. Homogenates were incubated at 4 °C with gentle agitation for 1 h, then centrifuged (Beckman Coulter Avanti® J25-I centrifuge) at 25,931 × *g* for 30 min, filtered through one layer of Miracloth™ (Merck Millipore), and finally re-centrifuged at 25,931 × *g* for 20 min. The pH of the clarified supernatants was adjusted to 8.4 with 1 M NaOH, and they were incubated at 4 °C with gentle agitation for approximately 48 h. The clarified plant extracts were centrifuged (Beckman Coulter Avanti® J25-I centrifuge) at 25,931 × *g* for 20 min, and the supernatants were loaded onto discontinuous Optiprep™ (Sigma Aldrich) iodixanol gradients (2 mL 50%, 2 mL 40%, 2 mL 30%, 2 mL 20%) diluted in bicine buffer. The gradients were re-centrifuged (Beckman Coulter Optima™ L-100 XP Ultracentrifuge) at 60,973 × *g* (22,400 rpm) for 3 h at 10°C using a Beckman SW 32 Ti rotor. Fractions of 500 µL were collected from the bottom of the centrifuge tubes and analysed by SDS-PAGE, western blotting and transmission electron microscopy (TEM) as described previously [8]. The purified VLP samples were quantitated by gel densitometry of Coomassie-stained polyacrylamide gels using GeneTools software (Syngene) and a bovine serum albumin (BSA) standard curve.

Immunogenicity of the BTV8 and chimaeric purified VLPs was tested in female guinea pigs. This study was approved by the Animal Ethics Committee at UCT (AEC020-023). Ten- to 12-week old animals were randomly separated into three groups of five guinea pigs each (n = 5). Group 1 was inoculated with mock antigen comprising the equivalent fraction extracted from negative control leaves and purified in a similar manner to the VLPs. Groups 2 and 3 were inoculated subcutaneously with 15 µg of BTV8 and chimaeric BTV1/8 VLPs, respectively, and mixed with 5% Montanide™ ISA 50 V2 adjuvant (Seppic). Pre-bleed serum was harvested prior to immunisation, and animals were boosted at 13 days post-immunisation. Final bleeds were collected on day 41 post-immunisation by cardiac puncture.

Indirect ELISA was conducted on sera to evaluate the vaccine immune responses using methods described by Stander et al. with some modifications [12]: ELISA plates were coated with 100 ng of plant-produced BTV8 or chimaeric BTV1/8 VLPs, pooled sera were diluted to 1:10,000 in TBS prior to loading into the wells containing the respective antigens against which they were raised, and goat anti-guinea pig IgG (whole molecule) alkaline-phosphatase-conjugated secondary antibody was diluted to 1:30,000 in 1x TBS. Statistical analysis was performed using GraphPad Prism version 9.3.1 (GraphPad, CA, USA). Error bars on the graphs represent the mean ± SEM (standard error of the mean) from three independent experiments with technical triplicates for each group. Differences in the response between groups were analyzed using Student’s two-tailed *t*-test. The significance threshold (*p*-value) was set at 5% (*p* = 0.05), with *p* < 0.05 representing a statistically significant result.

Sera from animals in groups 2 and 3 and a pooled sample from group 1 were assayed for virus neutralisation capability against BTV serotypes 1 and 8, using the microneutralisation method. Briefly, twofold serial dilutions of test sera at 25-µl volumes were made in tissue culture medium in wells of flat-bottomed microtitre plates. Approximately 100-300 TCID_50_ of reference BTV serotypes 1 and 8 were added in equal volumes to each well containing the test sera and gently mixed. The plates were incubated for 1 hour at 37 °C and 5% CO_2_, after which 10^4^ Vero cells (ATCC, Manassas, VA, USA) maintained in Dulbecco’s modified Eagle’s medium (DMEM) (Life Technologies, Carlsbad, CA, USA) containing 2% foetal bovine serum (FBS) (Sigma-Aldrich, Saint Louis, MO, USA) and 1× penicillin and 1× streptomycin (Gibco, New Yolk, NY, USA) were added to each well in a volume of 100 µl, and the plates were incubated for up to 7 days. Positive and negative antisera and cell controls and virus back-titration plates were set up per serotype accordingly, and the plates were observed daily for the development of a cytopathic effect (CPE), using an inverted microscope. Final test results were read when there was 75-100% CPE in the wells containing the test virus and in those containing the virus and negative control antisera and no CPE was observed in the cell control and in the wells containing virus and positive control antisera. Sera were considered to be specific for the reference BTV serotypes if they neutralised the virus in the test [13].

Co-infiltration with the BTV8 VP2-, VP3-, VP5-, and VP7-encoding constructs resulted in expression of all four proteins and assembly of VLPs, as expected from previous work [8]. To determine whether chimaeric BTV1/8 VLPs could be assembled in plants, the chimaeric BTV1/8 VP2 construct was co-infiltrated with the BTV8 VP3, VP5, and VP7 constructs.

After purification and fractionation, SDS-PAGE confirmed that the majority of the four BTV VP proteins in both samples accumulated at the interface of a 30-40% Optiprep™ gradient, and this was confirmed by western blotting (Supplementary Fig. S1A and B – VP2, 111 kDa; VP3, 103 kDa; VP5, 59 kDa; and VP7, 38 kDa), with the purest fractions ranging from 6 to 9 (BTV8) and 7 to 10 (BTV1/8) as determined by Coomassie blue staining (Supplementary Fig. S1C and D). Fractions from each of the samples were visualised using TEM, and the presence of VLPs was confirmed (Fig.1B). Core-like particles (CLPs) – consisting of VP3 and VP7 only – were also visible in both samples, possibly as a result of interruption of assembly upon harvesting of leaves. BTV8 and BTV1/8 samples had similar densities of particles and ratios of VLPs to CLPs. Purified samples were quantitated for inoculation of guinea pigs, with concentrations of approximately 35 mg per kg of fresh leaf weight (FLW).

BTV8 and BTV1/8 VLPs (15 µg of total BTV protein) were administered to guinea pigs using a prime-boost regimen to compare their ability to elicit serotype-specific immune responses. Indirect ELISA of pooled pre- and post-immune sera (1:10,000 dilution) for each of the VLP vaccines (groups 2 and 3) using VLPs as coating antigen showed a statistically significant difference (*p* < 0.01) in reactivity between the pre- and post-immunisation sera, whereas there was no difference between the sera from animals immunised with the negative control (group 1) (Fig. 2A). This confirmed that both VLP vaccines were capable of stimulating a specific response. A comparison between the antibody-specific responses of the two VLP vaccines using a post-immunisation serum (1:10,000 dilution) from each individual guinea pig in the vaccination groups showed that sera to both BTV8 and BTV1/8 VLPs (groups 2 and 3) yielded significantly higher (at least fivefold) mean OD_405_ values – 0.997 and 0.633, respectively – than the negative control group (OD_405_, 0.119) (Fig. 2B). Additionally, the BTV8 VLP vaccine induced a significantly stronger (1.5-fold) immune response than the chimaeric VLP vaccine (see *p*-values, Fig. 2B). This suggests that the chimaeric vaccine is not as potent as the BTV8 vaccine. For a strong neutralising antibody response, it is necessary for the VP2 protein and its epitopes to be displayed in the correct conformation. The chimaeric nature of the VP2 protein may have affected the epitope display or the interaction of VP2 with VP5, making it less effective in inducing an antibody response [14].

**Fig. 2:**
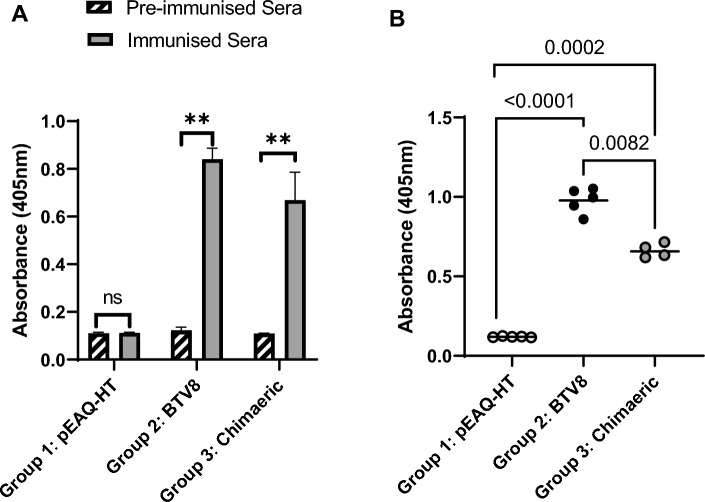
Indirect ELISA analysis of guinea pig serum. (A) Comparison of pooled sera from each vaccination group to determine the difference in antibody response before and after vaccination. Error bars represent the mean ± SEM from three independent experiments with technical triplicates for each group. ns, non-significant difference, where *p* > 0.05; **, significant difference, where *p* < 0.01. (B) Comparison of the post-immunisation antibody response between the three vaccination groups. Points represent the individual absorbance readings for each guinea pig averaged from three independent experiments with technical triplicates for each individual. Lines represent the group means. Student’s *t*-test was used to determine significance levels (p-values) between groups. The *y*-axis represents absorbance at 405 nm. The *x*-axis shows each vaccination group: group 1, negative control group immunised with mock pEAQ-*HT* antigen; group 2, immunised with BTV8 VLPs; group 3, immunised with BTV1/8 VLPs. Note: The data for group 3 consist of four points, as one animal was terminated early for medical and ethical reasons not pertaining to the study.

Serum neutralisation analysis was carried out on individual sera from each guinea pig. Sera from guinea pigs immunised with BTV8 VLPs (samples 1 to 5) neutralised BTV serotype 8 with virus neutralisation titres (VNTs) ranging from 1:40 to 1:160 (Table 1). Interestingly, these sera also neutralised BTV serotype 1, albeit with lower titres than those measured for BTV serotype 8. It is possible that this was due to cross-neutralisation between the VP2 domains of BTV serotypes 1 and 8. It has been shown previously that sera from animals infected with BTV8 could neutralise a cell-culture-adapted BTV1 strain [15]. In addition, Martinelle et al. [16] reported partial cross-reactivity between BTV1 and BTV8 in calves that were vaccinated with BTV8 and subsequently infected with BTV1. Sera from guinea pigs immunised with BTV1/8 chimaeric VLPs (samples 6 to 9) neutralised BTV serotype 1 with VNTs ranging from 1:20 to 1:40. The positive titres suggest that the substituted BTV1 VP2 domain may be responsible for conferring BTV1 specificity. The fact that sera from these same guinea pigs did not confer any BTV8 neutralisation capability further confirms this. This also suggests that the domains of BTV8 VP2 that are common to both BTV8 and BTV1/8 VLPs did not elicit a measurable VP2-specific response, thus confirming the importance of the tip domain as a major neutralisation epitope.

**Table 1:** Serum neutralisation titres against BTV serotypes 1 and 8. BTV8, wild-type VLPs; BTV1/8, chimaeric VLPs

Vaccine	Sample no.	BTV serotype 1 titre	BTV serotype 8 titre
BTV8	1	1:40	1:160
BTV8	2	1:20	1:80
BTV8	3	1:20	1:40
BTV8	4	1:20	1:80
BTV8	5	1:20	1:40
BTV1/8	6	1:40	neg
BTV1/8	7	1:20	neg
BTV1/8	8	1:40	neg
BTV1/8	9	1:20	neg
Mock	10	neg	neg

Having previously shown that BTV serotype 8 VLPs made in plants are immunogenic, are able to elicit serotype-specific neutralising antibodies, and are efficacious, this additional investigation showed that it is potentially possible to generate chimaeric VLPs targeting alternative serotypes that are also immunogenic and have neutralising capability. The retention of the serotype 8 backbone comprising VP3, VP5, and VP7 and substitution of the immunogenic tip domain of VP2 with that of a desired BTV serotype simplifies manipulation and overcomes the difficulties encountered with plant expression and/or assembly of all four serotype-specific VPs into VLPs. A vaccine cocktail of chimaeric VLPs of this nature could potentially be administered to animals to protect against multiple circulating serotypes. In addition, this vaccine design would be more accommodating for combatting outbreaks when the serotypes in circulation cannot be easily predicted prior to an outbreak.

## Supplementary Information

Below is the link to the electronic supplementary material.

Supplementary file1 (PPTX 515 kb)

Supplementary file2 (DOCX 15 kb)

## Data Availability

All datasets generated during and/or analysed during the current study are available from the corresponding author on reasonable request.
