# Supplementary material for: Chimaeric plant-produced bluetongue virus particles as potential vaccine candidates
Source: Arch Virol. 2023 Jun 13;168(7):179. doi: 10.1007/s00705-023-05790-x (PMC10264491; doi:10.1007/s00705-023-05790-x)
Supplement: Supplementary file 1 — Supplementary file1 (PPTX 515 kb) [file 705_2023_5790_MOESM1_ESM.pptx]

## Slide 1
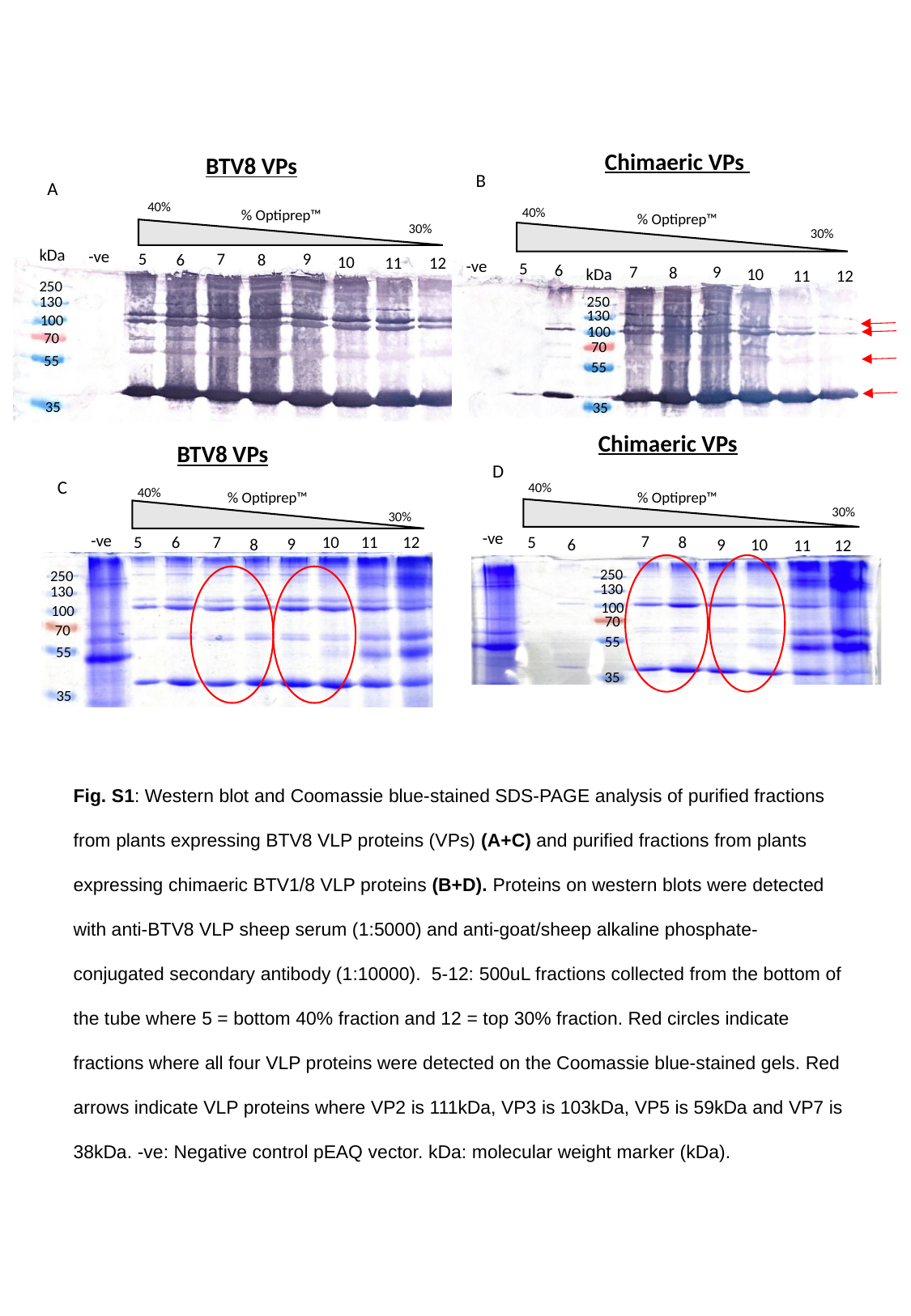

Chimaeric VPs
% Optiprep™
-ve
 5
6
9
 7
 8
10
11
12
250
130
100
70
55
35
B
kDa
40%
30%
BTV8 VPs
A
% Optiprep™
kDa
-ve
9
 7
 5
 8
6
10
11
12
250
130
100
70
55
35
40%
30%
Chimaeric VPs
BTV8 VPs
% Optiprep™
-ve
 7
10
6
11
 5
9
 8
12
250
130
100
70
55
35
C
40%
30%
D
40%
% Optiprep™
30%
-ve
 7
 5
 8
9
10
6
12
11
250
130
100
70
55
35
Fig. S1: Western blot and Coomassie blue-stained SDS-PAGE analysis of purified fractions from plants expressing BTV8 VLP proteins (VPs) (A+C) and purified fractions from plants expressing chimaeric BTV1/8 VLP proteins (B+D). Proteins on western blots were detected with anti-BTV8 VLP sheep serum (1:5000) and anti-goat/sheep alkaline phosphate-conjugated secondary antibody (1:10000). 5-12: 500uL fractions collected from the bottom of the tube where 5 = bottom 40% fraction and 12 = top 30% fraction. Red circles indicate fractions where all four VLP proteins were detected on the Coomassie blue-stained gels. Red arrows indicate VLP proteins where VP2 is 111kDa, VP3 is 103kDa, VP5 is 59kDa and VP7 is 38kDa. -ve: Negative control pEAQ vector. kDa: molecular weight marker (kDa).
