# Supplementary material for: Chimaeric plant-produced bluetongue virus particles as potential vaccine candidates
Source: Arch Virol. 2023 Jun 13;168(7):179. doi: 10.1007/s00705-023-05790-x (PMC10264491; doi:10.1007/s00705-023-05790-x)
Supplement: Supplementary file 2 — Supplementary file2 (DOCX 15 kb) [file 705_2023_5790_MOESM2_ESM.docx]

Table S1

GenBank accession numbers of the 37 BTV serotype 1 VP2 sequences aligned to generate consensus sequence tip domain.

|  | GenBank Accession number |
| --- | --- |
| 1 | MF384442.1 |
| 2 | MF384443.1 |
| 3 | MF384444.1 |
| 4 | MF384445.1 |
| 5 | MF384446.1 |
| 6 | MF384447.1 |
| 7 | MF384448.1 |
| 8 | MF384449.1 |
| 9 | MF384450.1 |
| 10 | MF384451.1 |
| 11 | MF384452.1 |
| 12 | MF384453.1 |
| 13 | MF384454.1 |
| 14 | MF384455.1 |
| 15 | MF384456.1 |
| 16 | MF384457.1 |
| 17 | MF384458.1 |
| 18 | MF384459.1 |
| 19 | MF384460.1 |
| 20 | MF384461.1 |
| 21 | MF384462.1 |
| 22 | MF384463.1 |
| 23 | MF384464.1 |
| 24 | MF384465.1 |
| 25 | MF384466.1 |
| 26 | MF384467.1 |
| 27 | MF384468.1 |
| 28 | MF384469.1 |
| 29 | MF384470.1 |
| 30 | MF384471.1 |
| 31 | MF384472.1 |
| 32 | MF384473.1 |
| 33 | KF664114.1 |
| 34 | KF664124.1 |
| 35 | FJ437557.1 |
| 36 | EU498674.1 |
| 37 | KY072170.1 |
